# Should my child be given antibiotics? A systematic review of parental decision making in rural and remote locations

**DOI:** 10.1186/s13756-024-01409-1

**Published:** 2024-09-19

**Authors:** Stephanie A. Marsh, Sara Parsafar, Mitchell K. Byrne

**Affiliations:** 1 School of Psychology, University of Wollongong, Wollongong, NSW Australia; 2 Discipline of Psychology, School of Arts and Sciences, The University of Notre Dame Australia, Sydney, NSW Australia

**Keywords:** Antibiotic use, Antimicrobial resistance, Parents, Children, Decision-making, Rural

## Abstract

**Background:**

The emergence and growth in antibiotic resistant bacteria is a critical public health problem exacerbated by the misuse of antibiotics. Children frequently succumb to illness and are often treated with antibiotic medicines which may be used improperly by the parent. There is limited evidence of the factors influencing parental decision-making about the use of antibiotics in low-resource contexts. The aim of this systematic review was to understand and describe how parents living in rural and remote locations make choices about their children’s antibiotic use.

**Method:**

The CINAHL, Web of Science, Medline, Scopus and Academic Search Premier databases were systematically searched from 31 January until 28 June in 2023. No date restrictions were applied and additional search methods were utilised to identify further studies that met inclusion criteria. Eligibility criteria included studies which reported on factors contributing to parental decisions about their children’s use of antibiotics in rural and remote settings. The Joanna Briggs Institute Critical Appraisal Checklists were employed to evaluate studies. Characteristics and findings were extracted from studies, and data was synthesised descriptively and presented in summary tables.

**Results:**

A total of 3827 articles were screened and 25 worldwide studies comprising of quantitative, qualitative and prospective designs were included in the review. Studies that reported the number of rural caregivers consisted of 12 143 participants. Data analysis produced six broad themes representing the mechanisms that influenced parents in their access and use of antibiotics: the child’s symptoms; external advice and influences; parent-related determinants; barriers to healthcare; access to antibiotics; and socio-demographic characteristics.

**Conclusions:**

A number of factors that influence parents’ prudent use of antibiotics in rural contexts were identified. In seeking to enhance appropriate use of antibiotics by parents in rural and remote settings, these determinants can serve to inform interventions. However, the identified studies all relied upon parental self-reports and not all studies reviewed reported survey validation. Further research incorporating validated measures and intervention strategies is required.

**Registration details:**

*Should my child be given antibiotics? A systematic review of parental decision making in rural and remote locations*; CRD42023382169; 29 January 2023 (date of registration). Available from PROSPERO.

**Supplementary Information:**

The online version contains supplementary material available at 10.1186/s13756-024-01409-1.

## Background

Antibiotics are critical in the treatment of infections caused by bacteria and can be lifesaving medicines in early life [1]. However, widespread and indiscriminate use of antibiotics is a significant contributor to the development of drug resistant pathogens, known as antimicrobial resistance (AMR) [2, 3]. AMR is increasing on a global scale, and currently accounts for approximately 700 000 deaths each year worldwide. This rate is predicted to increase exponentially to 10 million deaths by 2050, deepening the impact on health systems as infections become harder to treat [4]. Accordingly, the World Health Organization (WHO) has ranked AMR in the top ten global health threats [5]. Children are vulnerable to frequent bouts of illness [6] and are among the highest consumers of antibiotics [1, 7]. The United Nations Children’s Fund (UNICEF) has described AMR as “…perhaps the greatest threat to child survival and health of this generation” [8 p.2]. However, parental use of antibiotics with their children can be a key contributor to AMR. For example, despite the common experience of respiratory illness in childhood and the frequent use of antibiotics, only a small proportion of upper respiratory infections are of bacterial origin requiring antibiotic treatment [2].

The drivers of antibiotic overuse and misuse in children are multi-factorial and relate to both over-prescribing by health professionals [9] and the way that parents use antibiotics, such as autonomous practices [10] and failure to follow antibiotic treatment instructions [4]. Given that parents are the end-users and decide on behalf of the child how medication is obtained and used [11], understanding parent choices in their use of antibiotics is crucial to the determinants of inappropriate antibiotic use [12]. Parental decisions about antibiotic use with their children are influenced by a range of person and context variables. Previous systematic reviews have examined and quantified non-prescription antibiotic use in children [3, 13], describing parental knowledge about the use of antibiotics [2] and attitudes of parents about antibiotic prescribing in children as key drivers in antibiotic use [4]. Furthermore, systematic review findings indicate that residing in a rural location, and distance to healthcare, are associated with parents using antibiotics without consulting a doctor [13], a practice linked to the emergence of drug resistance [14]. This systematic review builds upon prior research and reviews by examining patterns in the decisional processes of parents living exclusively in rural communities around the world towards their use of antibiotics. This research also draws upon theoretical models explaining health-related behaviours to interpret and understand the review findings.

The mechanisms by which rurality and healthcare access influence parental decisions about antibiotic use are integral to understanding how parents in such locations can be supported to make more judicious decisions regarding the use of antibiotics with their children. Recent Australian research examining parents living in rural locations found that parental decisions about their children’s antibiotic use were influenced by fear of serious illness, and exacerbated by limited access to healthcare [15]. The influence of contextual factors is pertinent, as high rates of antibiotic resistant bacteria have been detected in children living in rural communities [16]. Drug resistant bacteria in children is especially concerning because of the contraindications of some antibiotics there are fewer options available to safely treat children [17].

Recent Australian research involving parents living in remote areas [15] provides some insight into how the context of rurality and healthcare access influence parental decisions. However, there are no systematic reviews which have examined the decision-making processes and influences of parents towards their children’s antibiotic use in rural, resource-limited settings. Understanding the drivers of parental behaviour in rural contexts can help to guide policy and target interventions to slow the growth of AMR. Thus, the objective of this review was to systematically describe the decision-making process of rural parents regarding their use of antibiotics. We sought to address the following research question: What factors influence the decisions of parents with children living in rural and remote locations in their use of antibiotics? Using the insights drawn from the recent Australian study [15] to facilitate search terms, we reviewed the international literature to identify factors influencing parents to initiate antibiotic therapy and the motivators of their antibiotic use behaviours.

## Methods

A pre-defined research protocol was registered with the International Prospective Register of Systematic Reviews (PROSPERO) prior to the commencement of the review. This research was conducted following the Preferred Reporting Items for Systematic Reviews and Meta-Analysis (PRISMA) 2020 guidelines to ensure transparency and accurate reporting [18]. No amendments were made to the protocol during the review process. (Further details of the completed PRISMA 2020 checklist and adherence to the statement can be found in Additional file 1).

### Eligibility criteria

Inclusion and exclusion criteria were pre-specified in the study protocol and applied during screening. Worldwide studies available in English were included, and no restrictions were placed on the publication period. Both peer-reviewed and grey literature were accepted. Studies were eligible if they provided data on the decision-making process of parents living in rural and remote locations in their use of antibiotics with their children (aged 0–18 years). This included an examination of all factors influencing parent decisions to use antibiotics, and factors contributing to how antibiotics were acquired and used. *Parents* were defined in accordance with the National Library of Medicine as: ‘persons functioning as natural, adoptive, or substitute parents’, such as caregivers [19]. *Factors* were described as circumstances, facts or influences contributing to parental decisions. We considered quantitative and qualitative designs, mixed methods, observational, prospective, systematic reviews, cross-sectional and longitudinal studies. Studies incorporating both rural and urban parents met inclusion criteria if there was clear delineation between urban and rural parent responses. If multiple medications were examined, or other population sub-groups were in the sample, these studies were included if the exclusive results were provided for antibiotics and parents. We excluded studies without data (i.e., editorials, protocol designs and letters) and intervention studies relating to antibiotic stewardship and treatment compliance. Studies that did not provide data on the decision-making process of parents towards their children’s antibiotic use, non-parent/caregiver samples and studies specifically examining other antimicrobial agents (antiviral, anti-fungal, anti-parasitic), or other medicines, were excluded. Studies based in urban and semi-urban settings were also excluded from the review.

### Search strategy

Five electronic databases were systematically searched: Web of Science; Scopus; EBSCOhost databases (Academic Search Premier, CINAHL and Medline). The search was performed from 31 January 2023 and monitored weekly through database alerts until 28 June 2023. A final update of each database was performed on 16 June 2023. Our search strategy included a combination of key words and Medical Subject Heading (MeSH) terms. Search terms were developed using a variation of the Population, Intervention, Comparison, Outcomes, Study design/Setting (PICOS) elements and were guided by prior study findings [15]. The search strategy was reviewed and refined by an academic librarian. Terms were separated into their synonym groups when combined in searches to build a multi-line search strategy, connected with Boolean connectors (AND/OR). A preliminary search was conducted using the search string to test for the identification of records. Search terms were adapted for use to account for changes in database symbols, or other search syntax, particular to a database. Database-specific filters were applied where available. (Details of the full search strategies for all databases are outlined in Additional file 2). To identify additional articles, the reference lists of studies eligible for full-text review and grey literature websites/search engines, were searched between 3 and 22 March 2023. Grey literature covered both accessible and inaccessible articles and included searching: Trove; Open Grey; OpenDOAR; NZresearch.org.nz; MedNar; Western Pacific Region Index Medicus; Clinical Trials Search Portal; Theses Canada; PsycINFO; and Google Scholar. A mix of key words and simplified search strings were used to account for differences in search interfaces after consultation with a research librarian. (Further details of the supplementary searches are provided in Additional file 2).

### Selection of studies

Records identified from electronic databases and other sources described were downloaded and stored in EndNote software version X9. All records were screened for duplication by the principal reviewer (SM) using EndNote tools and through a manual process of checking, identifying and removing duplicate records. The titles and abstracts of records were screened by SM using a pre-defined decision tree to guide judgements in the screening and selection of studies for inclusion in the review. The decision tree was a methodological approach used to assist the reviewers to stay organised and transparent in their approach and to ensure that the inclusion and exclusion of studies was based on predefined criteria to minimise the risk of bias in the review process. Fidelity checks were conducted by the other reviewers (MB and SP) on a random selection (10%) of included and excluded records. Discrepancies were resolved through team discussion, or referred to the third reviewer to determine study inclusion. Articles included in the next stage, underwent full-text evaluation by SM using a full-text decision tree tool to assess study eligibility. (Further details of both decision trees are available in Additional file 3). A random sample (10%) of full-text articles were reviewed by (MB and SP) to check decisions against the decision tree criteria. Inconsistencies in opinion were discussed until a consensus was reached, or when there was disagreement, the third reviewer made the final decision regarding study inclusion. During screening, one study investigator was emailed to confirm participant characteristics, to no avail. Subsequently, study inclusion was discussed amongst the researchers until agreement was attained.

### Data extraction

A standardised format was used to collect and enter study data into a Microsoft Excel spreadsheet using labelled columns. The initial extraction was performed by one reviewer (SM) and re-checked for accuracy. Subsequently, two other members of the research team (MB and SP) crossed-checked the extracted data. Findings were discussed and agreement was reached on the corresponding information. In one study, participants were described as ‘parents/carers of young children’ and the age range of the children was not specified [20]. However, we included this study based on the assumption that ‘young children’ was the same as pre-adolescents (primary school age and younger), given that parents were recruited through early childhood education services and playgroups. The following pre-specified data was collected from each eligible study, similar to the information extracted in prior reviews [4]: author name; year of publication; country; study aim; study setting/context; participant information (caregiver and child characteristics); study design and methodology; key findings (principal outcomes and results relevant to the current research); and study limitations. The primary outcome of interest was: the drivers/determinants of parents’ decisions towards the use of antibiotics with their children. Variables investigated included factors predisposing parents to use antibiotics (factors associated with use), and determinants of how antibiotics were accessed and used by parents (i.e., prescription/non-prescription use, asking prescribers for antibiotics, and antibiotic adherence behaviour). We considered any measure of parental decision-making, which was predominantly self-report.

### Critical appraisal

We assessed the possibility of bias and the methodological quality of studies included in the next phase of the review, using the Joanna Briggs Institute (JBI) critical appraisal checklists. JBI has shifted towards using the term ‘risk of bias’, notably for quantitative analytical designs, which does not include assessing quality constructs. However, different terminology remains, and is suitable when assessing qualitative or other types of research evidence [21]. The JBI tools were selected according to the design of each study assessed, and all items in the corresponding checklists were utilised. Subsequently, we employed the following four JBI checklists: studies reporting prevalence data; qualitative research; cohort studies; and analytical cross-sectional studies. Mixed-methods designs were appraised using the checklist reporting prevalence data, as well as the checklist for qualitative research. Two reviewers (SM and SP) independently assessed full-text studies (i.e., appraisals were performed by the principal reviewer and checked by SP). A proportion (10%) of randomly selected studies were assessed by the third reviewer (MB) using the JBI tools. Responses were recorded using the JBI forms and comments were added to support judgements, where necessary. Discrepancies in appraisals were managed through discussion between the reviewers to reach agreement.

A pre-determined JBI score of 60% or above was decided by the review team, which is indicative of moderate to high-quality studies [22] and has been applied in past reviews [3]. Inclusion of studies scoring low on JBI may jeopardise the validity of the review findings [23]. Thus, only studies reaching 60% or higher ‘yes’ results after discrepancies between reviewers had been resolved were included in the review. For mixed-methods designs, if the study met only partial inclusion, a reasoned and collaborative approach was employed by the research team [24] to consider the component that passed critical appraisal. We used the following score ranges to rate the potential risk of bias of individual studies: low risk of bias for studies achieving 70% or more ‘yes’ scores; moderate risk of bias for studies obtaining between 50 and 69% ‘yes’ scores (noting studies below 60% were excluded); and high risk of bias if the number of ‘yes’ scores was below 50% [25].

### Data synthesis

Data from individual studies included in the review was summarised by tabulating extracted information in a Microsoft excel spreadsheet for transparent reporting and to present a comparison of the findings. Studies were ordered within the spreadsheet by the year of publication to present the most recent research in the field to the earliest. A narrative synthesis of the findings was provided following the guidance of Popay and colleagues (2006) [26]. There was considerable heterogeneity in the study methods and approaches used, subsequently, a meta-analysis was not performed. However, the purpose of the review was to describe, rather than quantify, a process of decision-making. Key findings data was analysed to identify common patterns and differences across studies. This information was then synthesised and summarised descriptively in ‘Primary findings’ tables to address the research question. Comparisons were made between Australian and international research, where applicable. Primary findings were categorised into groups for the synthesis: (1) the process of decision-making to use or not use antibiotics i.e., influences on antibiotic use (2) factors contributing to specific behaviours with antibiotics identified i.e., non-prescription use of antibiotics, non-adherence to antibiotic treatment, requests for antibiotic prescriptions. Broader themes of the mechanisms underlying antibiotic use decisions were identified and categorised in the tables of primary findings. Data synthesis wasn’t categorised according to the age of the child as nearly all studies sampled children < 12yrs. The Grading of Recommendations Assessment, Development and Evaluation (GRADE) approach was not used to assess the certainty of evidence based on the limitation that all studies which are non-randomised control trials are rated as low evidence by GRADE [27].

## Results

### Study selection

**Fig. 1:**
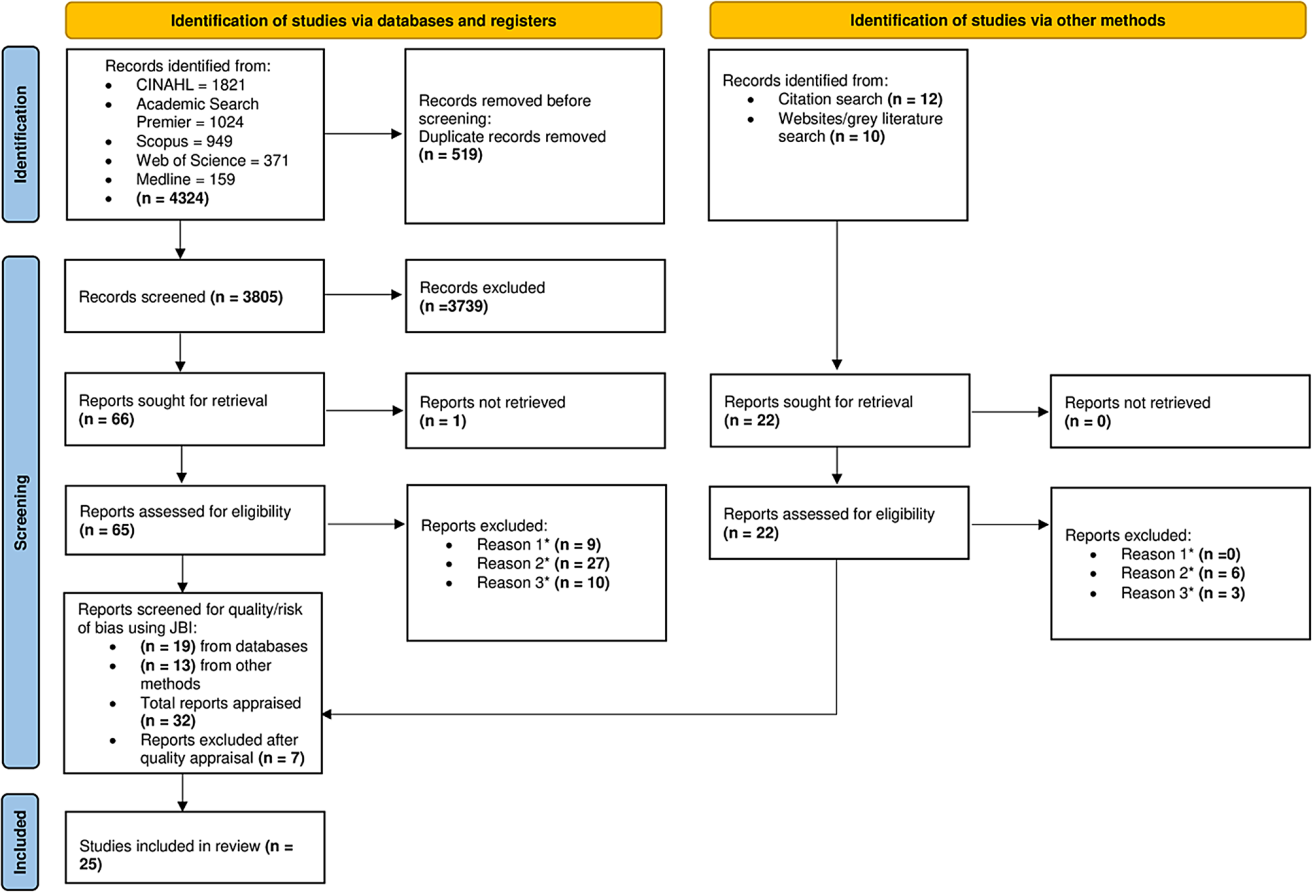
PRISMA 2020 flow diagram outlining the study selection process *Reasons for full-text exclusion: **Reason 1**: participant characteristics were not suitable; **Reason 2**: the study did not provide exclusive results for parents/caregivers, or antibiotics, or parents living in rural or remote areas; **Reason 3**: the study did not provide data on the decision-making process of rural parents towards their children’s antibiotic use

Electronic database searches and registers identified 4324 records and an additional 22 records were found via citation and grey literature search. No unpublished articles were identified. However, some grey literature sources searched both accessible and inaccessible articles. This assisted to uncover records that were not identified through the original electronic database search. After duplicate records were removed, 3805 titles and abstracts were screened. Most records excluded at this stage were unrelated to antibiotic use. Following this process and the identification of records from reference lists and other methods, 88 eligible full-text articles were sought. Only 1 report was unable to be retrieved in a full-text English language version. A total of 87 full-text articles were reviewed for eligibility. Reports were excluded for the following three reasons using the full-text decision tree tool: (1) participant characteristics were not suitable (2) the study did not provide exclusive results for parents/caregivers, or antibiotics, or parents living in rural or remote areas (3) the study did not provide data on the decision-making process of rural parents towards their children’s antibiotic use. Three studies appeared to meet inclusion criteria but were excluded because of difficulty delineating specific antibiotic use decisions for rural parents [28, 29], or we were unable to determine if both urban and rural sub-districts were amongst the findings [30]. During the final phase of screening, 32 studies were evaluated using the JBI checklists. Two mixed-methods studies that met partial inclusion were excluded from the review after team discussion. These were excluded because either the quantitative study was developed based on the qualitative research, which did not reach JBI score requirements, or the qualitative component met inclusion, but did not provide data relevant to the research question. After all appraisals were completed, 25 studies remained, and were included in the review. Figure 1 details the study selection process using the PRISMA 2020 flow diagram.

### Characteristics of studies

The 25 studies included in the review represented 14 countries across 6 continents: Asia (14); Africa (7); Central America (1); South America (1); Europe (1) and Australia (1). The highest number of studies (6) were conducted in Vietnam [31–36]. Publication periods spanned between 1996 and 2022. The design of 18 studies was quantitative [33, 34, 36–51], 6 were qualitative [20, 31, 35, 52–54] and 1 study was prospective [32]. Sample sizes varied from 13 to 3117 caregivers [48, 52] and up 4087 children [33] and depended on the study design. Cross-sectional studies had the largest samples [33, 37, 48] whereas qualitative designs with only a proportion of rural caregivers had the smallest [52]. Studies recruited either caregivers (parents, grandparents, other relatives, or carers), or specified parents or mothers. However, across all studies, most respondents were mothers. Only 2 studies included a small proportion of children > 12yrs [41, 51] and the majority sampled were children < 6yrs. One study stated that 2% of children were > 10yrs [45], and another specified ‘young children’ [20]. All other studies sampled children < 7yrs. Of the included studies: 18 studies examined the knowledge, attitudes or perceptions and behaviours of parents towards the use of antibiotics for their children [31, 32, 34, 37–39, 41–47, 49, 51–54]; 4 studies investigated parent opinions and practices towards childhood illnesses and health-seeking behaviours [20, 40, 48, 50]; and 3 studies examined patterns of drug use, including antibiotic use, amongst parents [33, 35, 36]. (Additional file 4 describes the characteristics and key findings of individual studies included in the review and includes all data used for analysis).

### Risk of bias quality assessment

The risk of bias for studies included in the review was classified as low for 16 studies [20, 31–36, 38, 41, 42, 44, 46, 51–54] and moderate for 9 studies [37, 39, 40, 43, 45, 47–50]. (A summary of the risk of bias assessments, including each domain/item assessed for all studies appraised is provided in Additional file 5). There is a possibility of recall and response bias in all of the studies, as each depended on parent self-reports of their experiences, perceptions and practices [4]. For most quantitative studies, it was unclear if the survey instrument had undergone full measurement validation. In some studies, pre-testing and pilot studies had been conducted (44%), indicating relative validation of the measurement tool [38, 40, 42–46, 48]. Other studies didn’t discuss instrument validation (33%) [33, 36, 37, 39, 41, 50]. Only a minority of studies (22%) reported the survey had been validated [34, 47, 49, 51]. All qualitative and prospective studies were considered low risk of bias [20, 31, 32, 35, 52–54]. However, some reporting elements were missing from the qualitative research: most did not include information about the researcher’s cultural or theoretical orientation (67%); and the majority of qualitative studies did not report the researcher’s philosophical perspective (67%).

Tables 1, 2, 3 and 4 synthesise the primary findings of the review and represent the finding of themes generated from data analysis. The themes and sub-themes describe the factors found to underlie parent decisions about antibiotic use. We established six themes that impacted the decisions of parents residing in rural locations in their use of antibiotics: the child’s symptoms; external advice and influences; parent-related determinants; barriers to healthcare; access to antibiotics; and socio-demographic characteristics. Mechanisms covered by these themes contributed to overuse and misuse behaviours related to the access and use of antibiotics in rural contexts and were tabulated according to the four core areas of investigation (i.e., influences on antibiotic use, non-prescription use of antibiotics, non-adherence to antibiotic treatment, requests for antibiotic prescriptions). The tables present data on when, why and how parents engaged with antibiotics in rural setting. (Further details of data coding and thematic development can be found in Additional file 6).

**Table 1:** Primary findings – Influences on antibiotic use

Themes and sub-themes	Studies	Location
**Child’s symptoms** ^**†**^		
***Nature of symptoms:***		
* Cough * Fever * Diarrhea * Colds/respiratory illness * Difficulty breathing * Ear infections	* [33, 34, 36, 42, 44, 48, 49, 52] * [32, 34, 39, 42–44, 54] * [33, 35–37, 39, 50] * [32, 35, 39, 40, 49, 52] * [33, 48] * [43]	* Vietnam, China, Cambodia, Nigeria, Tanzania^1^ * Vietnam, Indonesia, China, Srpska, Cambodia, Bangladesh^1^ * Vietnam, Bangladesh, Indonesia, Guatemala^1^ * Vietnam, Indonesia, Yemen, Nigeria, Tanzania^1^ * Vietnam, Nigeria^1^ * Srpska^2^
***Severity of symptoms:***		
* Seriousness of illness/longer duration * Combination of symptoms	* [31, 32, 37, 48] * [33, 39]	* Vietnam, Bangladesh, Nigeria^1^ * Vietnam, Indonesia^1^
**External advice and influences**		
***Prescriber advice:*** ***Advice of friends and family:***	* [31, 32, 39–43, 49, 51] * [40, 43, 49, 52, 54]	* Vietnam, Indonesia, Yemen, China, Srpska, Nigeria^1^ * Yemen, Srpska, Nigeria, Tanzania, Bangladesh^1^
**Socio-demographic characteristics**		
***Child age*** (conflicting findings):		
* >2-3yrs of age associated with antibiotic use. * Younger age associated with antibiotic use.	* [32, 42] * [39, 48]	* Vietnam, China^3^ * Indonesia, Nigeria^2^
**Parent-related determinants**		
(i.e., Parent/child distress caused by the pain of the injection contributed to parental hesitancy towards antibiotic use).	* [20]	* Australia^3^

**†**Symptoms were associated with antibiotic use (prescribed and unprescribed use) or parent expectations for antibiotics

^1^The risk of bias was rated low to moderate

^2^the risk of bias was rated moderate

^3^the risk of bias was rated low

**Table 2:** Primary findings – Non-prescription use of antibiotics

Themes and sub-themes	Studies	Location
**Child’s Symptoms**		
***Nature of symptoms:***		
* Cough * Diarrhea * Fever * Colds/respiratory illness * Difficulty breathing	* [33, 34, 36, 44, 48, 49, 52] * [33, 35–37, 50] * [32, 34, 44, 54] * [32, 35, 40, 49] * [33, 48]	* Vietnam, Cambodia, Nigeria, Tanzania^1^ * Vietnam, Bangladesh, Guatemala^1^ * Vietnam, Cambodia, Bangladesh^3^ * Vietnam, Yemen, Nigeria^1^ * Vietnam, Nigeria^1^
***Severity of symptoms*** (conflicting findings):		
* Children given unprescribed antibiotics for more severe/prolonged illness or multiple symptoms. * Children given unprescribed antibiotics for perceived minor illness.	* [33, 37] * [32, 35, 43, 51]	* Vietnam, Bangladesh^1^ * Vietnam, Srpska, China^1^
**Access to antibiotics**		
(i.e., availability to over-the-counter/affordable antibiotics or storing antibiotics in the home or sharing).	* [33–37, 41, 42, 46–54]	* Vietnam, Bangladesh, China, Uganda, Peru, Nigeria, Guatemala, Tanzania, Malawi^1^
**Barriers to healthcare**		
* Greater distance to travel: * Poor road conditions: * Lack of transportation: * Insufficient time, money or service availability:	* [36, 37, 46, 53, 54] * [36, 37] * [36, 37, 53] * [35, 43, 47, 49, 52, 54]	* Vietnam, Bangladesh, Uganda, Malawi^1^ * Vietnam, Bangladesh^1^ * Vietnam, Bangladesh, Malawi^1^ * Vietnam, Srpska, Peru, Nigeria, Tanzania, Bangladesh^1^
**External advice and influences**		
* Advice of others*(i.e., drug store suppliers, known health professionals, friends or relatives)*: * Social and cultural norms:	* [32, 34, 35, 44, 47–49, 52, 54] * [36, 46, 53, 54]	* Vietnam, Cambodia, Peru, Nigeria, Tanzania, Bangladesh^1^ * Vietnam, Uganda, Malawi, Bangladesh^3^
**Parent-related determinants**		
***Knowledge*** (conflicting findings):		
* Limited knowledge and unprescribed use. * No association between knowledge and unprescribed antibiotic use. * Knowledge of prescription requirements was associated with increased likelihood of parents storing leftover antibiotics.	* [34, 35, 37, 38, 40–42, 45, 51] * [47] * [42]	* Vietnam, Bangladesh, Nigeria, Yemen, China, Tanzania^1^ * Peru^2^ * China^3^
***Attitudes and beliefs*** (conflicting findings):		
* Parental beliefs about the efficacy of antibiotics and unprescribed use. * Parental attitudes the child’s condition is too minor to see a doctor and experience using prescribed antibiotics to treat similar symptoms. * Poor parental attitudes about the appropriate use of antibiotics. * Parental attitudes not related to unprescribed antibiotic use.	* [33–36, 49, 51, 52] * [43, 51] * [40, 45] * [47]	* Vietnam, Nigeria, China, Tanzania^1^ * Srpska, China^1^ * Yemen, Tanzania^2^ * Peru^2^
**Socio-demographic characteristics**		
***Parent education*** (conflicting findings):		
* Higher educated parents less likely to give unprescribed antibiotics. * Higher educated parents more likely to give unprescribed antibiotics.	* [38] * [32, 36]	* Nigeria^3^ * Vietnam^3^
***Parent age*** (conflicting findings):		
* Older parents/caregivers more likely to give unprescribed antibiotics. * Younger parents more likely to give unprescribed antibiotics.	* [41] * [38]	* China^3^ * Nigeria^3^
***Child age****(conflicting findings)*:		
* Increasing age of child associated with unprescribed antibiotic use. * Younger children more likely to be treated with antibiotics by the parent.	* [51] * [37, 48]	* China^3^ * Bangladesh, Nigeria^2^
***Multiple household members***:	* [41, 51]	* China^3^

^1^The risk of bias was rated low to moderate

^2^the risk of bias was rated moderate

^3^the risk of bias was rated low

**Table 3:** Primary findings – Non-adherence to antibiotic treatment

Themes and sub-themes	Studies	Location
**Parent-related determinants**		
***Attitudes and beliefs:***		
* Child’s symptoms improved * Concern of potential harmful effects * Lack of improvement in symptoms	* [35, 41, 42, 51, 53, 54] * [35, 54] * [53]	* Vietnam, China, Malawi, Bangladesh^3^ * Vietnam, Bangladesh^3^ * Malawi^3^
***Knowledge:***		
* Low awareness of AMR and side effects * Difficulties understanding the medicine regime.	* [34, 35, 40, 42] * [20, 53]	* Vietnam, Yemen, China^1^ * Australia, Malawi^3^
***Forgetting***:	* [20]	* Australia^3^
**External advice and influences**		
* Social and cultural norms: * Palatability/child refusal: * Lack of access to refrigeration to store antibiotic medicine:	* [53] * [20] * [20]	* Malawi^3^ * Australia^3^ * Australia^3^
**Barriers to healthcare**		
*Greater distance to travel:	* [53]	* Malawi^3^

^1^The risk of bias was rated low to moderate

^2^the risk of bias was rated moderate

^3^the risk of bias was rated low

**Table 4:** Primary findings – Requests for antibiotic prescriptions

Themes and sub-themes	Studies	Location
**Parent-related determinants**		
***Attitudes and beliefs***:		
*Attitudes it is appropriate to ask prescribers for antibiotics. *Attitudes prescribers should acquiesce to parental expectations for antibiotics.	* [51] * [47]	* China^3^ * Peru^2^

^1^The risk of bias was rated low to moderate

^2^the risk of bias was rated moderate

^3^the risk of bias was rated low

*Note:* Bold text in Tables 1, 2, 3 and 4 represents the themes and sub-themes

Table 1 describes the factors that motivated parents to use or not use antibiotics with their children.

Table 2 details the predictors of parental use of antibiotics without medical guidance.

Table 3 outlines the factors that influenced parents to cease their child’s antibiotic therapy early and not complete a full treatment course.

Lastly, Table 4 indicates the reasons parents placed demand on doctors to prescribe antibiotics for their child.

## Discussion

The findings of this review suggest that six themes underpinned the decisions of parents living in rural and remote settings towards their use of antibiotic medicines. The first theme was the *‘child’s symptoms’*, which represented findings concerning the nature and severity of illness. Our results highlight that parents perceived antibiotics as appropriate in the treatment of a variety of symptoms exhibited by their children including cough, fever, diarrhea, respiratory illness, breathing impairment, and ear infections. The nature of the child’s symptoms both motivated parental expectations for the provision of antibiotics and influenced antibiotic use, including autonomous practices (without reference to medical advice), which has been substantiated in prior reviews [13]. In more than half of the studies, parents used antibiotics autonomously to treat their children’s cough, diarrhea, fever, colds/respiratory infection and breathing difficulties. Cough and fever were the symptoms associated most with parental expectations to use antibiotics, and antibiotic use in general, including both prescribed and unprescribed use. However, when only non-prescription antibiotic use was examined, children with fever received unprescribed antibiotics in fewer studies than those with cough and diarrhea symptoms. This finding might be explained by research in China which reported that while parents were most concerned about fever and cough symptoms, they were less likely to use antibiotics to treat fever without consulting a doctor [41]. The severity of the child’s illness, duration and number of symptoms influenced antibiotic use and expectations for the child to receive antibiotics [31–33, 37, 39, 48], which is supported by previous research findings [4, 15]. It is likely that parental use of prescribed antibiotics was influenced by the prescriber’s decisions and advice [31, 32, 39], highlighting the importance of the opinions of others in decision-making. However, in relation to unprescribed use, parents treated their children with antibiotics for perceived minor illness in four studies [32, 35, 43, 51] and in two studies when the illness was more severe or multiple symptoms were reported [33, 37]. This finding may reflect that parents might try to seek medical advice for their child when the illness is believed to be serious, but appear to use antibiotics autonomously for a range of symptoms and severity levels when medical advice is less available.

The second theme that emerged from our analysis was *‘external advice and influences’.* This theme relates to findings that parents were influenced by the opinions and advice of others when making decisions about the use of antibiotics with their children. As expected, prescriber advice informed parental understanding of when antibiotics were required in a number of studies [31, 32, 39–43, 49, 51]. In two studies, even when parents perceived antibiotics were not necessary, they adhered to medical recommendations to treat their child with antibiotics [31, 32], highlighting the value placed on guidance from prescribers. Information from doctors about antibiotics has been identified as an important source of knowledge for parents in past research [13]. However, parents also considered the advice of friends and family, such as those with more experience, or ‘decision makers’ regarding their child’s treatment needs and the use of antibiotics [40, 43, 49, 52, 54]. We found that seeking advice from others contributed to parental decisions to use non-prescription antibiotics with their children. In nine studies where unprescribed antibiotics were used, parents obtained treatment advice from drug suppliers, health professionals living in their community, friends and relatives [32, 34, 35, 44, 47–49, 52, 54]. Additionally, social and cultural norms influenced autonomous antibiotic use and non-adherence behaviours. For example, parents acted in accordance with the views of others [53, 54], were influenced by pharmaceutical promotions [36] and obtained antibiotics from people in their network [46]. Findings in respect to non-adherence behaviours highlight that external factors, such as palatability and lack of refrigeration influenced early discontinuation of the child’s antibiotic course in an Australian study [20]. This highlights that some reasons for non-adherence were child-related or influenced by socio-economic factors.

The third theme identified from this review was *‘parent-related determinants’*. This theme reflects findings that knowledge, attitudes/beliefs and cognitive factors contributed to parental behaviours with antibiotics across the four core areas of investigation. A lack of parental knowledge and confusion regarding antibiotic use [20, 34, 35, 40, 42, 53], and beliefs about symptom improvement [35, 41, 42, 51, 53, 54] mostly accounted for non-adherence decisions. One study identified that parents pressured prescribers for prescriptions because they perceived it was appropriate to ask for antibiotics [51]. This finding underscores the influence of attitudinal (a right to receive a prescription) or knowledge (lack of understanding of the need for antibiotics) factors on behaviour. In relation to unprescribed antibiotic use, limited knowledge about the indications for use or the risks of use was identified in numerous studies where parents used antibiotics autonomously [34, 35, 37, 38, 40–42, 45, 51]. There was some inconsistency with this finding, with other studies reporting no association between parental knowledge and unprescribed antibiotic use [47] or observing that having adequate knowledge of prescription requirements was associated with increased likelihood of retaining leftovers [42]. Nonetheless, research did indicate that knowledge played an important role in the appropriate use of antibiotics, which has been supported by past reviews [2, 13]. However, possessing knowledge does not necessarily ensure responsible behaviours with antibiotics [2], as other factors appear to also drive autonomous practices [47]. For example, strong beliefs regarding the efficacy of antibiotics were discussed in a number of studies where non-prescription antibiotic use was prevalent [33–36, 49, 51, 52]. Of these, four studies discussed that antibiotics were perceived as a cure-all [33, 35, 36, 52], highlighting firm beliefs about antibiotics as curative drugs used to treat a range of illnesses, which likely influenced antibiotic use decisions. These results might explain why parents used antibiotics to treat many symptoms of childhood illness irrespective of the microbial source. Parental attitudes that the child’s condition is too minor to see a doctor and prior experience using prescription antibiotics to treat a similar illness, may explain parents self-treating symptoms perceived to be minor.

The results of this review identified *‘barriers to healthcare’* as a further theme. This theme includes findings of the difficulties experienced by parents in accessing health facilities in rural settings. Poor access to formal healthcare was a barrier to appropriate antibiotic use and predominantly impacted parents administering antibiotics to their children without consulting a doctor, consistent with previous research [13]. In the current review, ten studies reported on the specific mechanisms of limited access to healthcare believed to contribute to the non-prescription use of antibiotics. Insufficient time, money or service availability [35, 43, 47, 49, 52, 54], greater distance to travel [36, 37, 46, 53, 54], lack of transportation [36, 37, 53] and poor road conditions [36, 37] were challenges which influenced autonomous practices with antibiotics. Living greater than 5 miles [37] or 5 km [46] from the nearest hospital or health facility was associated with parents using unprescribed antibiotics with their children in two studies. Living far from healthcare facilities also provided the impetus to store leftover antibiotics as a strategy to manage resource limitations [53]. In seven studies citing poor access to healthcare, parents reported to obtain treatment advice from drug suppliers, family, friends from a health background [35, 47, 49, 52, 54] or parents shared antibiotics between their personal network [46, 53]. Analysing the information in the current review does not determine whether lack of access to healthcare caused parents to seek advice from others. However, separate to a predilection to listen to non-medical advice, when there are impediments to accessing healthcare, parents may utilise other available resources to assist with decision-making, or adopt strategies to gain access to antibiotics.

The fifth theme established from data analysis was *‘access to antibiotics’.* This theme relates to findings that availability of unprescribed antibiotics (i.e., over-the-counter sales, storing or sharing antibiotics) enabled autonomous use by parents. The availability of over-the-counter antibiotics at drug stores was discussed in almost half the studies as a contributor to non-prescription use [33–37, 47–52, 54]. In five studies, purchasing antibiotics without a prescription and storing antibiotics and leftovers in the home were associated with parents using antibiotics autonomously [36, 41, 42, 47, 51]. Antibiotic sharing also contributed to parents using antibiotics without consulting a doctor [46, 52, 53]. In a number of studies, drug stores were described as easily accessible in terms of distance, affordability or extended operating hours and may be negotiable in price, quantity or brand [35–37, 48–50]. It is important to note that in some settings it was reported that unregulated drug stores were the only feasible avenue for the community to access medicines and healthcare [54]. In nine studies reporting barriers to the formal health system, parents accessed antibiotics via non-prescription sale or by sharing or storing antibiotics [35–37, 46, 47, 49, 52–54]. The findings indicate that access to unprescribed antibiotics is a strong predictor of antibiotic use. Healthcare challenges appear to exacerbate autonomous behaviours resulting in parents deciding to seek more available treatment pathways.

The final theme of this review was *‘socio-demographic characteristics’*, which includes variables associated with antibiotic use and misuse, such as the child and parents’ age, parent education, and household composition. There were no clear patterns that emerged from socio-demographic factors, which is consistent with previous research [13]. In two studies it was observed that children over 2-3yrs of age had a higher likelihood of using antibiotics [32, 42], and increasing age of the child was associated with non-prescription antibiotic use in one study [51]. Studies in Australia and Vietnam indicate that parents may try to safeguard their young children from antibiotics [15, 32]. However, in contrast, other research found that younger children had the highest rates of antibiotic use [39, 48] and were more likely to be treated with unprescribed antibiotics [37]. In a Nigerian study it was opined that younger children may have received more antibiotics because of parental perceptions that illness is more serious in early life [48]. In the case of prescribed antibiotic use, we are unable to determine from our analysis of some studies if the age of the child impacted the parents’ decision to seek antibiotics, or if healthcare providers were inclined to prescribe more antibiotics based on the child’s age [32, 39].

With respect to non-prescription use of antibiotics, the parent’s age and level of education also produced conflicting findings. For example, in Nigeria, mothers with higher levels of education were more aware of the risks of misuse and were less inclined to self-treat with antibiotics [38]. On the contrary, studies in Vietnam observed more highly educated mothers were prone to autonomous antibiotic use [32, 36] and reported confidence in when to initiate antibiotics [36]. The mixed results produced for parent/child features and antibiotic use may reflect the number of different countries, cultures and their values. Cultural awareness and sensitivity should be considered when planning interventions to meet the needs of the target population. Nonetheless, having multiple children/household members was found to be associated with parents using unprescribed antibiotics in two Chinese studies [41, 51]. This finding has been supported in past reviews [13] and suggests that increased experience with using antibiotics may enhance confidence to self-treat.

### Theoretical models

The various themes that emerged from the analysis can be interpreted with reference to theories explaining health-related behaviours. Based on the results of this review, the most comprehensive explanation of parental behaviours with antibiotics is provided by the Theory of Planned Behaviour (TPB) and Self-Determination Theory (SDT). The TPB posits that three key constructs influence intention to perform a behaviour: *attitude* about the behaviour; *subjective norms* that others approve of the behaviour; and *perceptions of control* that the behaviour can be facilitated [55]. One or a combination of these variables facilitates an understanding of parental choices towards their children’s antibiotic use in rural settings. For example, the data suggests strong beliefs about the effectiveness of antibiotics in the treatment of numerous symptoms and favourable attitudes towards autonomous antibiotic use are likely predictors of antibiotic use behaviours. Associated with attitudes, deficits in antimicrobial knowledge likely contributed to the formation of parental attitudes about antibiotic use, although knowledge did not always motivate autonomous behaviour. Attitudes and beliefs were also found to contribute to non-adherence decisions and requests for antibiotic prescriptions. Our findings also indicate that advice and assent from others, subjective norms, including both medical and non-medical connections, contributed to antibiotic use and autonomous use. Relatedly, social pressure and cultural factors were also found to play a role in the non-adherence decisions of Malawian parents [53]. Perceived behavioural control to act appropriately and obtain medical guidance from a prescriber was impeded by cost, time, distance to services and limited healthcare options. While ease of availability to unregulated antibiotics facilitated antibiotic use and increased behavioural control over autonomous use decisions as a strategy to manage healthcare barriers.

Alternatively, the SDT model provides an alternative framework to understand why parents might initiate antibiotic therapy without consulting a doctor. SDT proposes that people have three core psychological needs: *autonomy* (i.e., the need for choice in their behaviour); *competence* (i.e., the need to feel capable and effective in influencing favourable outcomes); and *relatedness* (i.e., the feeling of connectedness, being understood and belonging with others). When the health context supports people in meeting these needs, people are increasingly motivated to be autonomous in their actions and maintain their health behaviours [56]. The data from this study suggests that unregulated access to antibiotics and support received from others in the social environment in the decision-making process towards autonomous antibiotic use, facilitates and maintains behavioural autonomy with antibiotics. Parental beliefs about the efficacy and potency of antibiotics in treating multiple symptoms likely influences feelings of competency in aiding their child’s recovery from illness by using antibiotics. Feelings of competency may be maintained by knowledge deficiencies about the risks and indications for antibiotic use. Furthermore, widespread use of over-the-counter antibiotics and acceptance of storing and sharing practices among households, likely supports feelings of relatedness and normalises behaviours utilised to access antibiotics and manage healthcare difficulties.

### Summary and limitations

This review uncovered six themes representing factors which contributed to when, why and how parents in rural and remote locations accessed and used antibiotics. These insights may assist in the development of programs to facilitate the appropriate use of antibiotics in rural and remote settings. By identifying the bespoke drivers of antibiotic use by parents within context of place and culture, interventions focussing on knowledge, attitudes, social norms, and behavioural competency in the management of children’s health needs can be developed. Such bespoke interventions necessitate measurement of the drivers of target parent behaviours using reliable and valid strategies.

A number of limitations are acknowledged in this research. This systematic review was limited to studies in English. However, grey literature and reference lists were searched without date restrictions, and a range of study designs were included in order to identify as much research as possible, which was a strength of this study. Care was taken to systematically categorise studies into themes and sub-themes. Nevertheless, we acknowledge this might introduce some degree of subjectivity to data analysis. Although the review was worldwide, limited Australian studies were identified that met the inclusion criteria. This presents a need for further research in rural and remote settings of Australia to investigate the appropriate use of antibiotics on larger samples of parents. Future reviews may also consider a comparison of urban and rural parent samples to ascertain any key differences/similarities of note in how decisions are formed. Lastly, survey validation procedures of studies included in this review were underreported. This highlights an opportunity for future research to report these processes more transparently to enhance the robustness of the findings.

## Conclusions

This study identified a number of determinants motivating parents in rural and remote areas to use antibiotics and factors which influenced their practices. Nearly all studies identified parents treating their children with non-prescription antibiotics, which was the most common misuse practice noted. Overuse and misuse of antibiotics is a critical driver in selecting and facilitating the development of drug resistant bacteria in communities. Accordingly, the results of this review give emphasis to several mechanisms that enabled antibiotic use as well as obstacles that prevented parents from making more optimal decisions about the use of antibiotics. The way in which these mechanisms inter-relate to influence the decisions and behaviour of parents should be considered when developing bespoke interventions to contain the impact of AMR in rural, low-resource contexts.

## Electronic supplementary material

Below is the link to the electronic supplementary material.

Additional file 1: Completed PRISMA 2020 checklist (.docx)

Additional file 2: Search strategy for all databases and supplementary searches (.docx)

Additional file 3: Decision tree tools used for data screening (.docx)

Additional file 4: Summary of study characteristics of included studies and all data used for analysis (.xls)

Additional file 5: JBI risk of bias quality assessments (.docx)

Additional file 6: Details of data coding/analysis and thematic development (.docx)

## Data Availability

All data generated or analysed during this study are included in this published article [and its supplementary information files].

## Declarations

### Authors’ information

MB is co-founder of the Wollongong Antimicrobial Resistance Research Alliance (WARRA) [57] and has authored several publications traversing issues in AMR. MB is Adjunct Associate Professor with the School of Psychology at the University of Wollongong. SP is a Psychology Lecturer and the Course Coordinator of Psychology Honours, and has authored several systematic reviews. SM is a PhD Student and a Registered Psychologist with experience in Health Psychology.

## Abbreviations

AMR: Antimicrobial resistance
WHO: World Health Organization
UNICEF: United Nations Children’s Fund
PROSPERO: International Prospective Register of Systematic Reviews
PRISMA: Preferred Reporting Items for Systematic Reviews and Meta-Analysis
MeSH: Medical Subject Headings
PICOS: Population, Intervention, Comparison, Outcomes, Study design/Setting
JBI: Joanna Briggs Institute
GRADE: Grading of Recommendations Assessment, Development and Evaluation
ARI: Acute Respiratory Tract Infection
PPMVs: Patent and Proprietary Medicine Vendors
TPB: Theory of Planned Behaviour
SDT: Self-Determination Theory
