# Supplementary material for: Should my child be given antibiotics? A systematic review of parental decision making in rural and remote locations
Source: Antimicrob Resist Infect Control. 2024 Sep 19;13:105. doi: 10.1186/s13756-024-01409-1 (PMC11412025; doi:10.1186/s13756-024-01409-1)
Supplement: Supplementary file 2 — Additional file 2: Search strategy for all databases and supplementary searches (.docx) [file 13756_2024_1409_MOESM2_ESM.docx]

**Additional file 2**

**Full search strategy for all databases and supplementary searches**

**Search terms:**

- Antibiotic OR antibiotics OR antibiotic use OR anti-bacterial agents OR self-medication OR behavior OR non-adherence
- Parent OR parents OR caregivers OR mothers OR fathers
- Child OR children OR infant OR baby OR toddler OR paediatric OR kids OR youth OR adolescent OR teenager
- Decisions OR decision making OR motivation OR influence OR action OR determinants OR attitude OR beliefs OR fear OR social norms OR risk aversion OR limited access
- Rural population OR rural OR remote OR rurality OR villages OR isolated OR regional OR distance OR distant

**Web of Science:**

- antibiotic* or anti*bacterial agents or self*medication or behavio$r or non*adherence

AND

- parent* or care* or mother* or father*

AND

- child* or bab* or toddler or adolescent or youth or infant or p$ediatric or kids or teenager

AND

- decision* or motivation or influenc* or action or determinants or attitude or beliefs or fear or social norms or risk aversion or limited access

AND

- rural population or rural or remote or rurality or villages or isolated or regional or distan*

**Filter applied:** Antibiotics & Antimicrobials

**Medline:**

- antibiotic* or anti*bacterial agents or self*medication or behavio?r or non*adherence

AND

- parent* or care* or mother* or father*

AND

- child* or bab* or toddler or adolescent or youth or infant or p?ediatric or kids or teenager

AND

- decision* or motivation or influenc* or action or determinants or attitude or beliefs or fear or social norms or risk aversion or limited access

AND

- rural population or rural or remote or rurality or villages or isolated or regional or distan*

**Filter applied:** anti-bacterial agents

**Academic Search Premier:**

- antibiotic* or anti*bacterial agents or self*medication or behavio?r or non*adherence

AND

- parent* or care* or mother* or father*

AND

- child* or bab* or toddler or adolescent or youth or infant or p?ediatric or kids or teenager

AND

- decision* or motivation or influenc* or action or determinants or attitude or beliefs or fear or social norms or risk aversion or limited access

AND

- rural population or rural or remote or rurality or villages or isolated or regional or distan*

**Filter applied:** Peer Reviewed

**CINAHL:**

- antibiotic* or anti*bacterial agents or self*medication or behavio#r or non*adherence

AND

- parent* or care* or mother* or father*

AND

- child* or bab* or toddler or adolescent or youth or infant or p#ediatric or kids or teenager

AND

- decision* or motivation or influenc* or action or determinants or attitude or beliefs or fear or social norms or risk aversion or limited access

AND

- rural population or rural or remote or rurality or villages or isolated or regional or distan*

**Filter applied:** Nil

**Scopus:**

- antibiotic* or anti*bacterial agents or self*medication or *behavior or non*adherence

AND

- parent* or care* or mother* or father*

AND

- child* or bab* or toddler or adolescent or youth or infant or *pediatric or kids or teenager

AND

- decision* or motivation or influenc* or action or determinants or attitude or beliefs or fear or social norms or risk aversion or limited access

AND

- rural population or rural or remote or rurality or villages or isolated or regional or distan*

**Filter applied:** Nil. One line search across ‘all fields’.

**Other websites/search engines**

- **Trove:** trove.nla.gov.au
- **Searched:** ‘parent antibiotic use’, ‘parent use of antibiotics with their children’
- **Open Grey:** [www.opengrey.eu](http://www.opengrey.eu)
- **Searched:** ‘parent antibiotic use’, ‘parent use of antibiotics with their children’, ‘antibiotic use’
- **OpenDOAR:** <https://v2.sherpa.ac.uk/opendoar/>
- **Searched:** ‘parent antibiotic use’, ‘parent use of antibiotics with their children’, ‘antibiotic use’
- **NZresearch.org.nz:** <https://nzresearch.org.nz/>
- **Searched:** ‘parent antibiotic use’, ‘parent antibiotic use rural’, ‘antibiotic use’, ‘child antibiotic use’
- **MedNar:** <https://mednar.com/mednar/desktop/en/search.html>
- **Searched:** ‘parent use of antibiotics with their children rural’ ‘parent survey antibiotic use’
- **Western Pacific Region Index Medicus:** [www.wprim.org](http://www.wprim.org)
- **Searched:** ‘parent use of antibiotics with their children rural’, ‘parent use of antibiotics with their children’, ‘parent antibiotic use’
- **Clinical Trials Search Portal:** <https://trialsearch.who.int/>
- **Searched:** ‘parent antibiotic use’
- **Theses Canada**: [www.bac-lac.gc.ca/eng/services/theses/Pages/theses-canada.aspx](http://www.bac-lac.gc.ca/eng/services/theses/Pages/theses-canada.aspx)
- **Searched:** ‘parent use of antibiotics with their children rural’
- **PsycINFO**: Medline search strategy
- **Searched:** ‘Dissertations’
- **Google Scholar**: <https://scholar.google.com.au/>
- **Searched:** ‘parent use of antibiotics with their children rural’, ‘parent antibiotic use rural:pdf’, ‘Antimicrobial resistance, caregivers, paediatrics, antibiotics’, ‘Use of antibiotics for children rural community’
