# Supplementary material for: Should my child be given antibiotics? A systematic review of parental decision making in rural and remote locations
Source: Antimicrob Resist Infect Control. 2024 Sep 19;13:105. doi: 10.1186/s13756-024-01409-1 (PMC11412025; doi:10.1186/s13756-024-01409-1)
Supplement: Supplementary file 3 — Additional file 3: Decision tree tools used for data screening (.docx) [file 13756_2024_1409_MOESM3_ESM.docx]

**Additional file 3**

**Decision Tree Tools**

| **Inclusion Criteria** | | | | **Exclusion** |  |
| --- | --- | --- | --- | --- | --- |
| **1. Is the title and abstract in English?**  **INCLUDE:**  English language studies  **EXCLUDE:** Non-English language studies  ↓  Yes  **2. Is the type of study suitable?** **INCLUDE:** Quantitative and qualitative designs, mixed methods, observational, prospective, systematic reviews, longitudinal and cross-sectional studies about antibiotics.  **EXCLUDE:** Studies that do not relate to antibiotic use or studies solely investigating non-antibiotic medicines. Studies not using data, such as editorials, protocol designs and letters. Intervention studies relating to antibiotic stewardship or treatment compliance.  ↓  Yes | | | | → No  → No | **Paper is excluded from the review.** |
| **3. Does the study examine parents in their use of antibiotic medicines for their children?** **INCLUDE:** Parent/caregiver antibiotic use studies. Studies investigating parents in their use of multiple types of medication, including antibiotics, and studies incorporating both parents and other population sub-groups will be included. Studies that do not specify who the consumers or population sub-groups in the sample are will be included at this stage for further review. **EXCLUDE:** studies solely investigating non-parent/caregiver samples.  ↓  Yes | | | | → No |  |
| **4. Is the study based in a rural or remote context?**  **INCLUDE:** Studies based in rural or remote locations. If there is uncertainty about whether the study is based in a rural or remote location, the study will be included at this stage. Studies specifying a proportion of parents in the sample live in rural or remote areas and comparison studies between urban and rural parents will be included for further review of eligibility.  **EXCLUDE:** Studies based in urban and semi-urban settings. | | | | → No |  |
| ↓  Yes | | | |  |  |
|  | |  |  |  |  |
|  | |  |  |  |  |
|  |  |  |  |  |  |
|  |  |  |  |  |  |
| **Reports for Retrieval** | | | |  |  |

**Title and Abstract Decision Tree**

**Full-Text Decision Tree**

| **Inclusion Criteria** | | **Exclusion** |  |
| --- | --- | --- | --- |
| **1. Are the participant characteristics suitable?**  **INCLUDE:** studies with participants who are parents/caregivers of children aged between 0 and 18yrs.  **EXCLUDE:** studies of parents of children >18yrs of age. Non-parent/caregiver samples, such as prescribers, the general public, animals, plants and agriculture will be excluded.  ↓  Yes  **2. Does the study provide exclusive results for parents/caregivers?** **INCLUDE:** studies that have both parents/caregivers and other population sub-groups in the sample will be included, if the exclusive results are provided for parents/caregivers of children.  **EXCLUDE:** studies that do not differentiate between the results of parents/caregivers and other population sub-groups will be excluded.  ↓  Yes | | → No  → No | **Paper is excluded from the review.** |
| **3. Does the study provide exclusive results for antibiotic use?** **INCLUDE:** parent/caregiver antibiotic use studies. Studies examining parents in their use of multiple types of medication, including antibiotics, or comparison studies between antibiotic and non-antibiotic medication use, will be included, if the exclusive results for antibiotics are provided.  **EXCLUDE:** studies examining parents in their use of other types of medication will be excluded. Studies examining multiple types of medications, including antibiotics, as well as comparison studies between antibiotic and non-antibiotic medicines, will be excluded, if the exclusive results for antibiotics are not provided.  ↓  Yes | | → No |  |
| **4. Does the study provide exclusive results for parents living in rural and remote areas?**  **INCLUDE:** Rural and remote based studies. Comparison studies between rural and urban parents or studies incorporating a proportion of both rural and urban parents in the sample, will be included, if there is clear delineation between urban and rural parent responses.  **EXCLUDE:** studies identified as being based in urban or semi-urban settings. Comparison studies between rural and urban parents or studies incorporating a proportion of both rural and urban parents in the sample, will be excluded, if there isn’t clear delineation between urban and rural parent responses. | | → No |  |
| ↓  Yes | |  |  |
| **5. Does the study provide data on the decision-making process of rural parents towards child antibiotic use?** **INCLUDE:** studies that provide data on the circumstances, facts or influences contributing to decisions about whether parents use antibiotics with their children and how antibiotics are used by parents. **EXCLUDE:** studies that do not provide data on the circumstances, facts or influences contributing to parental decisions about whether they use antibiotics with their children and how antibiotics are used by parents. There must be sufficient data to clearly delineate antibiotic use decisions for rural/remote parents. | | → No |  |
| ↓  Yes | |  |  |
|  |  |  |  |
|  |  |  |  |
| Include the paper in the review. | |  |  |
