# Supplementary material for: Should my child be given antibiotics? A systematic review of parental decision making in rural and remote locations
Source: Antimicrob Resist Infect Control. 2024 Sep 19;13:105. doi: 10.1186/s13756-024-01409-1 (PMC11412025; doi:10.1186/s13756-024-01409-1)
Supplement: Supplementary file 5 — Additional file 5: JBI risk of bias quality assessments (.docx) [file 13756_2024_1409_MOESM5_ESM.docx]

**Additional file 5**

**JBI risk of bias quality assessment**

| **Studies** | **JBI’s appraisal checklist for studies reporting prevalence data** | | | | | | | | |  |  |
| --- | --- | --- | --- | --- | --- | --- | --- | --- | --- | --- | --- |
|  | **Q1** | **Q2** | **Q3** | **Q4** | **Q5** | **Q6** | **Q7** | **Q8** | **Q9** | **Yes%** | **Risk^a^** |
| Akinlade et al. (2015) | Yes | Yes | Yes | Yes | Yes | UC | UC | Yes | Yes | 78% | Low |
| Collett et al. (1999) | UC | No | No | Yes | UC | UC | Yes | Yes | Yes | 44% | High |
| Ding et al. (2015) | Yes | No | Yes | Yes | Yes | UC | Yes | Yes | Yes | 78% | Low |
| Graham et al. (2016) - mixed methods | UC | Yes | No | Yes | UC | UC | Yes | Yes | UC | 44% | High |
| Halfvarsson et al. (2000) - mixed methods | Yes | No | No | Yes | Yes | UC | Yes | Yes | Yes | 67% | Moderate |
| Hoan et al. (2009) | Yes | Yes | Yes | Yes | Yes | No | Yes | Yes | UC | 78% | Low |
| Mijovic et al. (2022) | Yes | Yes | Yes | Yes | Yes | UC | No | Yes | UC | 67% | Moderate |
| Mutagonda et al. (2022) | Yes | Yes | Yes | Yes | Yes | UC | UC | Yes | UC | 67% | Moderate |
| O’Connor et al. (2001) | No | No | No | Yes | Yes | No | Yes | Yes | Yes | 56% | Moderate |
| Okumura et al. (2002) | Yes | Yes | UC | Yes | Yes | No | Yes | Yes | Yes | 78% | Low |
| Paredes et al. (2022) | No | No | No | Yes | Yes | Yes | Yes | Yes | Yes | 67% | Moderate |
| Uneke et al. (2021) | Yes | Yes | UC | Yes | Yes | Yes | UC | Yes | UC | 67% | Moderate |
| Al-Noban & Elnimeiri (2022) | Yes | Yes | Yes | Yes | Yes | UC | No | Yes | UC | 67% | Moderate |
| Larsson et al. (2000) | Yes | Yes | UC | Yes | Yes | Yes | Yes | Yes | UC | 78% | Low |
| Miyazaki et al. (2020) | Yes | Yes | No | Yes | Yes | UC | Yes | Yes | Yes | 78% | Low |
| Nyeko et al. (2022) | Yes | Yes | Yes | Yes | Yes | UC | No | Yes | Yes | 78% | Low |
| Salako et al. (2001) | Yes | Yes | UC | Yes | Yes | UC | Yes | Yes | UC | 67% | Moderate |
| Ahmed et al. (2018) | Yes | Yes | Yes | Yes | Yes | No | UC | Yes | UC | 67% | Moderate |
| Alkaff et al. (2019) | Yes | Yes | No | Yes | Yes | UC | UC | Yes | Yes | 67% | Moderate |
| Bhalla et al. (2019) | UC | Yes | Yes | Yes | Yes | No | UC | UC | UC | 44% | High |
| Cheng et al. (2019) | Yes | Yes | Yes | Yes | Yes | No | Yes | Yes | Yes | 89% | Low |
| Kaljee et al. (2011) | Yes | Yes | UC | Yes | No | UC | Yes | Yes | UC | 56% | Moderate |
| Van der Stuyft et al. (1996) | Yes | Yes | UC | Yes | Yes | No | Yes | Yes | UC | 67% | Moderate |

**Note: ^a^** The risk of bias was rated as high if the number of ‘yes’ scores was below 50%, moderate risk of bias for studies obtaining between 50 to 69% ‘yes’ scores (noting studies below 60% were excluded), and low risk of bias when the study reached more than 70% ‘yes’ scores. ‘UC’ indicates unclear. **Q1-9** of JBI’s critical appraisal checklist for studies reporting prevalence data [**Q1:** Was the sample frame appropriate to address the target population? **Q2:** Were study participants sampled in an appropriate way? **Q3:** Was the sample size adequate? **Q4:** Were the study subjects and the setting described in detail? **Q5:** Was the data analysis conducted with sufficient coverage of the identified sample? **Q6:** Were valid methods used for the identification of the condition? **Q7:** Was the condition measured in a standard, reliable way for all participants? **Q8:** Was there appropriate statistical analysis? **Q9:** Was the response rate adequate, and if not, was the low response rate managed appropriately?

| **Studies** | **JBI’s appraisal checklist for qualitative research** | | | | | | | | |  |  |  |
| --- | --- | --- | --- | --- | --- | --- | --- | --- | --- | --- | --- | --- |
|  | **Q1** | **Q2** | **Q3** | **Q4** | **Q5** | **Q6** | **Q7** | **Q8** | **Q9** | **Q10** | **Yes%** | **Risk^a^** |
| Amgarth-Duff et al. (2019) | UC | Yes | Yes | Yes | Yes | Yes | Yes | Yes | Yes | Yes | 90% | Low |
| Graham et al. (2016) - mixed methods | No | Yes | Yes | Yes | Yes | No | No | No | Yes | Yes | 60% | Moderate |
| Halfvarsson et al. (2000) - mixed methods | UC | Yes | Yes | UC | UC | No | No | No | UC | UC | 20% | High |
| Hoa et al. (2007) | UC | Yes | Yes | Yes | Yes | No | Yes | Yes | Yes | Yes | 80% | Low |
| King et al. (2018) | UC | Yes | Yes | Yes | Yes | Yes | Yes | Yes | Yes | Yes | 90% | Low |
| Lucas et al. (2019) | Yes | Yes | Yes | Yes | Yes | No | No | Yes | Yes | Yes | 80% | Low |
| Le et al. (2011) | UC | Yes | Yes | Yes | Yes | No | No | Yes | Yes | Yes | 70% | Low |
| Emgard et al. (2022) | Yes | Yes | Yes | Yes | Yes | No | Yes | Yes | Yes | Yes | 90% | Low |

**Note: ^a^** The risk of bias was rated as high if the number of ‘yes’ scores was below 50%, moderate risk of bias for studies obtaining between 50 to 69% ‘yes’ scores (noting studies below 60% were excluded), and low risk of bias when the study reached more than 70% ‘yes’ scores. ‘UC’ indicates unclear. **Q1-10** of JBI’s critical appraisal checklist for qualitative research [**Q1**: Is there congruity between the stated philosophical perspective and the research methodology? **Q2**: Is there congruity between the research methodology and the research question or objectives? **Q3:** Is there congruity between the research methodology and the methods used to collect data? **Q4:** Is there congruity between the research methodology and the representation and analysis of data? **Q5:** Is there congruity between the research methodology and the interpretation of results? **Q6:** Is there a statement locating the researcher culturally or theoretically? **Q7:** Is the influence of the researcher on the research, and vice- versa, addressed? **Q8:** Are participants, and their voices, adequately represented? **Q9:** Is the research ethical according to current criteria or, for recent studies, and is there evidence of ethical approval by an appropriate body? **Q10:** Do the conclusions drawn in the research report flow from the analysis, or interpretation, of the data?].

| **Studies** | **JBI’s appraisal checklist for cohort studies** | | | | | | | | |  |  |  |  |
| --- | --- | --- | --- | --- | --- | --- | --- | --- | --- | --- | --- | --- | --- |
|  | **Q1** | **Q2** | **Q3** | **Q4** | **Q5** | **Q6** | **Q7** | **Q8** | **Q9** | **Q10** | **Q11** | **Yes%** | **Risk^a^** |
| Hoa et al. (2011) | Yes | Yes | UC | Yes | Yes | Yes | UC | Yes | Yes | No | Yes | 73% | Low |

**Note: ^a^** The risk of bias was rated as high if the number of ‘yes’ scores was below 50%, moderate risk of bias for studies obtaining between 50 to 69% ‘yes’ scores (noting studies below 60% were excluded), and low risk of bias when the study reached more than 70% ‘yes’ scores. ‘UC’ indicates unclear. **Q1-11** of JBI’s critical appraisal checklist for cohort studies [**Q1:** Were the two groups similar and recruited from the same population? **Q2:** Were the exposures measured similarly to assign people to both exposed and unexposed groups? **Q3:** Was the exposure measured in a valid and reliable way? **Q4:** Were confounding factors identified? **Q5:** Were strategies to deal with confounding factors stated? **Q6:** Were the groups/participants free of the outcome at the start of the study (or at the moment of exposure)? **Q7:** Were the outcomes measured in a valid and reliable way? **Q8:** Was the follow up time reported and sufficient to be long enough for outcomes to occur? **Q9:** Was follow up complete, and if not, were the reasons to loss to follow up described and explored? **Q10:** Were strategies to address incomplete follow up utilized? **Q11:** Was appropriate statistical analysis used?].

| **Studies** | **JBI’s appraisal checklist for analytical cross-sectional studies** | | | | | | | | |  |
| --- | --- | --- | --- | --- | --- | --- | --- | --- | --- | --- |
|  | **Q1** | **Q2** | **Q3** | **Q4** | **Q5** | **Q6** | **Q7** | **Q8** | **Yes%** | **Risk^a^** |
| Quagliarello et al. (2003) | No | UC | Yes | Yes | No | No | UC | Yes | 38% | High |
| Yu et al. (2014) | UC | Yes | Yes | Yes | Yes | Yes | Yes | Yes | 88% | Low |

**Note: ^a^** The risk of bias was rated as high if the number of ‘yes’ scores was below 50%, moderate risk of bias for studies obtaining between 50 to 69% ‘yes’ scores (noting studies below 60% were excluded), and low risk of bias when the study reached more than 70% ‘yes’ scores. ‘UC’ indicates unclear. **Q1-8** of JBI’s critical appraisal checklist for analytical cross-sectional studies [**Q1:** Were the criteria for inclusion in the sample clearly defined? **Q2:** Were the study subjects and the setting described in detail? **Q3:** Was the exposure measured in a valid and reliable way? **Q4:** Were objective, standard criteria used for measurement of the condition? **Q5:** Were confounding factors identified? **Q6:** Were strategies to deal with confounding factors stated? **Q7:** Were the outcomes measured in a valid and reliable way? **Q8:** Was appropriate statistical analysis used?].

**Studies excluded based on JBI appraisal:**

1. Collett CA, Pappas DE, Evans BA, Hayden GF. Parental knowledge about common respiratory infections and antibiotic therapy in children. South Med J. 1999;92(10):971-6.
2. Graham K, Sinyangwe C, Nicholas S, King R, Mukupa S, Källander K, et al. Rational use of antibiotics by community health workers and caregivers for children with suspected pneumonia in Zambia: a cross-sectional mixed methods study. BMC Public Health. 2016;16(1):897.
3. Halfvarsson J, Heijne N, Ljungman P, Ham MN, Holmgren G, Tomson G. Knowing when but not how!--mothers' perceptions and use of antibiotics in a rural area of Viet Nam. Trop Doct. 2000;30(1):6-10.
4. O'Connor S, Rifkin D, Yang YH, Wang JP, Levine OS, Dowell SF. Physician control of pediatric antimicrobial use in Beijing, China, and its rural environs. Pediatr Infect Dis J. 2001;20(7):679-84.
5. Bhalla K, Gupta A, Nanda S, Mehra S, Verma S. Parental knowledge and common practices regarding acute respiratory infections in children admitted in a hospital in rural setting. J Family Med Prim Care. 2019;8(9):2908-11.
6. Kaljee LM, Anh DD, Minh TT, Huu Tho L, Batmunkh N, Kilgore PE. Rural and urban Vietnamese mothers utilization of healthcare resources for children under 6 years with pneumonia and associated symptoms. J Behav Med. 2011;34(4):254-67.
7. Quagliarello AB, Parry CM, Hien TT, Farrar JJ. Factors associated with carriage of penicillin-resistant Streptococcus pneumoniae among Vietnamese children: a rural-urban divide. J Health Popul Nutr. 2003;21(4):316-24.
