# Supplementary material for: Should my child be given antibiotics? A systematic review of parental decision making in rural and remote locations
Source: Antimicrob Resist Infect Control. 2024 Sep 19;13:105. doi: 10.1186/s13756-024-01409-1 (PMC11412025; doi:10.1186/s13756-024-01409-1)
Supplement: Supplementary file 6 — Additional file 6: Details of data coding/analysis and thematic development (.docx) [file 13756_2024_1409_MOESM6_ESM.docx]

**Additional file 6**

**Coding and data analysis**

*Development of data patterns:*

| **Influences on antibiotic use** | | | |
| --- | --- | --- | --- |
| **Indicators for use** | **Studies** | **Location** | **Risk of bias** |
| **Child's symptoms:**  * Cough  * Fever  * Diarrhea  * Colds/respiratory illness  * Difficulty breathing  * Ear infections  (**Note:** symptoms were associated with antibiotic use or parent expectations for antibiotics) | * [33], [34], [36], [42], [44], [48], [49], [52]  * [32], [34], [39], [42], [43], [44], [54]  * [33], [35], [36], [37], [39], [50]  * [32], [35], [39], [40], [49], [52]  * [33], [48]  * [43] | * Vietnam, China, Cambodia, Nigeria, Tanzania  * Vietnam, Indonesia, China, Srpska, Cambodia, Bangladesh  * Vietnam, Bangladesh, Indonesia, Guatemala  * Vietnam, Indonesia, Republic of Yemen, Nigeria, Tanzania  * Vietnam, Nigeria  * Republic of Srpska | * Low to moderate  * Low to moderate  * Low to moderate  * Low to moderate  * Low to moderate  * Moderate |
| * Severity of child's illness/longer illness duration  * Combination of symptoms | * [31], [32], [37], [48]  * [33], [39] | * Vietnam, Bangladesh, Nigeria  * Vietnam, Indonesia | * Low to moderate  *Low to moderate |
| * Prescriber advice | * [31], [32], [39], [40], [41], [42], [43], [49], [51] | * Vietnam, Indonesia, Yemen, China, Srpska, Nigeria | * Low to moderate |
| * Advice of friends and family | * [40], [43], [49], [52], [54] | * Yemen, Srpska, Nigeria, Tanzania, Bangladesh | *Low to moderate |
| **Child age** (conflicting findings):  * >2-3yrs of age associated with antibiotic use  * Younger age associated with antibiotic use | * [32], [42]  * [39], [48] | * Vietnam, China  * Indonesia, Nigeria | * Low  * Moderate |
| *Parent/child distress caused by the pain of the injection contributed to parental hesitancy to use antibiotics. | * [20] | * Australia | *Low |
| **Non-prescription use of antibiotics** | | | |
| **Reasons** | **Studies** | **Location** | **Risk of bias** |
| **Child's Symptoms:**  * Cough  * Diarrhea  * Fever  * Colds/respiratory illness  * Difficulty breathing | * [33], [34], [36], [44], [48], [49], [52]  * [33], [35], [36], [37], [50]  * [32], [34], [44], [54]  * [32], [35], [40], [49]  * [33], [48] | * Vietnam, Cambodia, Nigeria, Tanzania  * Vietnam, Bangladesh, Guatemala  * Vietnam, Cambodia, Bangladesh  * Vietnam, Republic of Yemen, Nigeria  * Vietnam, Nigeria | * Low to moderate  * Low to moderate  *Low  * Low to moderate  * Low to moderate |
| **Severity of child's illness** (conflicting findings):  * Children given unprescribed antibiotics for more severe/prolonged illness or multiple symptoms.  * Children given unprescribed antibiotics for perceived minor illness. | * [33], [37]  * [32], [35], [43], [51] | *Vietnam, Bangladesh  * Vietnam, Republic of Srpska, China | * Low to moderate  * Low to moderate |
| * **Availability and access** to over-the-counter/affordable antibiotics or storing antibiotics in the home or sharing. | * [33], [34], [35], [36], [37], [41], [42], [46], [47], [48], [49], [50], [51], [52], [53], [54] | * Vietnam, Bangladesh, China, Uganda, Peru, Nigeria, Guatemala, Tanzania, Malawi | *Low to moderate |
| **Lack of access to healthcare/rurality**  (Specific factors discussed include):  * Greater distance to travel  * Poor road conditions  * Lack of transportation  * Insufficient time, money or service availability | * [35], [36], [37], [41], [43], [46], [47], [49], [51], [53]  * [36], [37], [46], [53], [54]  * [36], [37]  * [36], [37], [53]  * [35], [43], [47], [49], [52], [54] | * Vietnam, Bangladesh, China, Republic of Srpska, Uganda, Peru, Nigeria, Malawi  * Vietnam, Bangladesh, Uganda, Malawi  * Vietnam, Bangladesh  * Vietnam, Bangladesh, Malawi  * Vietnam, Republic of Srpska, Peru, Nigeria, Tanzania, Bangladesh | *Low to moderate  * Low to moderate  * Low to moderate  * Low to moderate  * Low to moderate |
| * **Advice of others** (drug store suppliers, known health professionals, friends or relatives)  * Social and cultural norms | * [32], [34], [35], [44], [47], [48], [49], [52], [54]  * [36], [46], [53], [54] | * Vietnam, Cambodia, Peru, Nigeria, Tanzania, Bangladesh  * Vietnam, Uganda, Malawi, Bangladesh | * Low to moderate  * Low |
| **Knowledge** (conflicting findings):  * Limited knowledge and unprescribed use.  * No association between knowledge and unprescribed antibiotic use.  * Knowledge of prescription requirements was associated with increased likelihood of parents storing leftover antibiotics. | * [34], [35], [37], [38], [40], [41], [42], [45], [51]  * [47]  * [42] | * Vietnam, Bangladesh, Nigeria, Yemen, China, Tanzania  * Peru  * China | * Low to moderate  * Moderate  * Low |
| **Attitudes and beliefs** (conflicting findings):  * Parental beliefs about the efficacy of antibiotics.  * Parental attitudes the child's condition is too minor to see a doctor and experience using prescribed antibiotics to treat similar symptoms.  * Poor parental attitudes about the appropriate use of antibiotics.  * Parental attitudes not related to unprescribed antibiotic use. | * [33], [34], [35], [36], [49], [51], [52]  * [43], [51]  * [40], [45]  * [47] | * Vietnam, Nigeria, China, Tanzania  * Republic of Srpska, China  * Republic of Yemen, Tanzania  * Peru | * Low to moderate  * Low to moderate  * Moderate  *Moderate |
| **Socio-demographic factors** (conflicting findings):  * Higher educated parents less likely to give unprescribed antibiotics.  * Higher educated parents more likely to give unprescribed.  * Older parents/caregivers more likely to give unprescribed antibiotics.  * Younger parents more likely to give unprescribed antibiotics.  * Increasing age of child associated with unprescribed antibiotic use.  * Younger children more likely to be treated with antibiotics by the parent.  * Multiple household members | * [38]  * [32], [36]  * [41]  * [38]  * [51]  * [37], [48]  * [41], [51] | * Nigeria  * Vietnam  * China  * Nigeria  * China  * Bangladesh, Nigeria  *China | * Low  * Low  * Low  * Low  * Low  * Moderate  *Low |
| **Non-adherence to treatment** | | | |
| **Reasons** | **Studies** | **Location** | **Risk of bias** |
| * Child's symptoms improved | * [35], [41], [42], [51], [53], [54] | * Vietnam, China, Malawi, Bangladesh | * Low |
| * Concern of potential harmful effects | * [35], [54] | * Vietnam, Bangladesh | * Low |
| * Low awareness of AMR and side effects | * [34], [35], [40], [42] | * Vietnam, Republic of Yemen, China | * Low to moderate |
| * Difficulties understanding the medicine regime | * [20], [53] | * Australia, Malawi | *Low |
| * Poor access to healthcare | * [53] | * Malawi | * Low |
| * Social and cultural norms | * [53] | * Malawi | * Low |
| *Lack of improvement in symptoms | * [53] | * Malawi | * Low |
| * Forgetting | * [20] | * Australia | * Low |
| * Palatability/child refusal | * [20] | * Australia | * Low |
| * Lack of access to refrigeration to store antibiotic medicine | * [20] | * Australia | * Low |
| **Requests for antibiotic prescriptions** | | | |
| **Reasons** | **Studies** | **Location** | **Risk of bias** |
| *Attitudes it is appropriate to ask prescribers for antibiotics.  *Attitudes doctors should acquiesce to parental expectations for antibiotics. | * [51]  * [47] | * China  * Peru | * Low  * Moderate |

*Data patterns/contributing factors were then synthesised and divided into six broader themes identified:*

1. **Child's symptoms** (sub-themes: nature of symptoms and severity of symptoms)
2. **External advice and influences** (sub-themes: prescriber advice; advice of friends and family; advice of others; social and cultural norms; palatability/child refusal; lack of access to refrigeration to store antibiotic medicine)
3. **Parent-related determinants** (sub-themes: parent distress, knowledge; attitudes and beliefs; forgetting)
4. **Socio-demographic characteristics** (sub-themes: child age; parent education; parent age; multiple household members)
5. **Access to antibiotics**
6. **Barriers to healthcare** (sub-themes: greater distance to travel; poor road conditions; lack of transportation; insufficient time/money/or service availability)

*Primary findings*

| **Influences on antibiotic use** | | | |
| --- | --- | --- | --- |
| **Themes and sub-themes** | **Studies** | **Location** | **Risk of bias** |
| **Child's symptoms^†^**  ***Nature of symptoms:***  * Cough  * Fever  * Diarrhea  * Colds/respiratory illness  * Difficulty breathing  * Ear infections  ***Severity of symptoms:***  * Seriousness of illness/longer duration  * Combination of symptoms | * [33], [34], [36], [42], [44], [48], [49], [52]  * [32], [34], [39], [42], [43], [44], [54]  * [33], [35], [36], [37], [39], [50]  * [32], [35], [39], [40], [49], [52]  * [33], [48]  * [43]  * [31], [32], [37], [48]  * [33], [39] | * Vietnam, China, Cambodia, Nigeria, Tanzania  * Vietnam, Indonesia, China, Srpska, Cambodia, Bangladesh  * Vietnam, Bangladesh, Indonesia, Guatemala  * Vietnam, Indonesia, Republic of Yemen, Nigeria, Tanzania  * Vietnam, Nigeria  * Republic of Srpska  * Vietnam, Bangladesh, Nigeria  * Vietnam, Indonesia | * Low to moderate  * Low to moderate  * Low to moderate  * Low to moderate  * Low to moderate  * Moderate  * Low to moderate  *Low to moderate |
| **External advice and influences**  ***Prescriber advice:***  ***Advice of friends and family:*** | * [31], [32], [39], [40], [41], [42], [43], [49], [51]  * [40], [43], [49], [52], [54] | * Vietnam, Indonesia, Yemen, China, Srpska, Nigeria  * Yemen, Srpska, Nigeria, Tanzania, Bangladesh | * Low to moderate  *Low to moderate |
| **Socio-demographic characteristics**  ***Child age*** (conflicting findings):  * >2-3yrs of age associated with antibiotic use  * Younger age associated with antibiotic use | * [32], [42]  * [39], [48] | * Vietnam, China  * Indonesia, Nigeria | * Low  * Moderate |
| **Parent-related determinants**  (i.e., Parent/child distress caused by the pain of the injection contributed to parental hesitancy about antibiotic use). | * [20] | * Australia | *Low |
| **Non-prescription use of antibiotics** | | | |
| **Themes and sub-themes** | **Studies** | **Location** | **Risk of bias** |
| **Child's Symptoms**  ***Nature of symptoms:***  * Cough  * Diarrhea  * Fever  * Colds/respiratory illness  * Difficulty breathing  ***Severity of symptoms*** (conflicting findings)***:***  * Children given unprescribed antibiotics for more severe/prolonged illness or multiple symptoms.  * Children given unprescribed antibiotics for perceived minor illness. | * [33], [34], [36], [44], [48], [49], [52]  * [33], [35], [36], [37], [50]  * [32], [34], [44], [54]  * [32], [35], [40], [49]  * [33], [48]  * [33], [37]  * [32], [35], [43], [51] | * Vietnam, Cambodia, Nigeria, Tanzania  * Vietnam, Bangladesh, Guatemala  * Vietnam, Cambodia, Bangladesh  * Vietnam, Yemen, Nigeria  * Vietnam, Nigeria  *Vietnam, Bangladesh  * Vietnam, Srpska, China | * Low to moderate  * Low to moderate  *Low  * Low to moderate  * Low to moderate  * Low to moderate  * Low to moderate |
| **Access to antibiotics**  (i.e., availability to over-the-counter/affordable antibiotics or storing antibiotics in the home or sharing). | * [33], [34], [35], [36], [37], [41], [42], [46], [47], [48], [49], [50], [51], [52], [53], [54] | * Vietnam, Bangladesh, China, Uganda, Peru, Nigeria, Guatemala, Tanzania, Malawi | *Low to moderate |
| **Barriers to healthcare**  **** Greater distance to travel:***  **** Poor road conditions:***  **** Lack of transportation:***  **** Insufficient time, money or service availability:*** | * [36], [37], [46], [53], [54]  * [36], [37]  * [36], [37], [53]  * [35], [43], [47], [49], [52], [54] | * Vietnam, Bangladesh, Uganda, Malawi  * Vietnam, Bangladesh  * Vietnam, Bangladesh, Malawi  * Vietnam, Republic of Srpska, Peru, Nigeria, Tanzania, Bangladesh | * Low to moderate  * Low to moderate  * Low to moderate  * Low to moderate |
| **External advice and influences**  **** Advice of others*** *(i.e., drug store suppliers, known health professionals, friends or relatives):*  **** Social and cultural norms:*** | * [32], [34], [35], [44], [47], [48], [49], [52], [54]  * [36], [46], [53], [54] | * Vietnam, Cambodia, Peru, Nigeria, Tanzania, Bangladesh  * Vietnam, Uganda, Malawi, Bangladesh | * Low to moderate  * Low |
| **Parent-related determinants**  ***Knowledge*** (conflicting findings):  * Limited knowledge and unprescribed use.  * No association between knowledge and unprescribed antibiotic use.  * Knowledge of prescription requirements was associated with increased likelihood of parents storing leftover antibiotics.  ***Attitudes and beliefs*** (conflicting findings):  * Parental beliefs about the efficacy of antibiotics and unprescribed use.  * Parental attitudes the child's condition is too minor to see a doctor and experience using prescribed antibiotics to treat similar symptoms.  * Poor parental attitudes about the appropriate use of antibiotics.  * Parental attitudes not related to unprescribed antibiotic use. | * [34], [35], [37], [38], [40], [41], [42], [45], [51]  * [47]  * [42]  * [33], [34], [35], [36], [49], [51], [52]  * [43], [51]  * [40], [45]  * [47] | * Vietnam, Bangladesh, Nigeria, Republic of Yemen, China, Tanzania  * Peru  * China  * Vietnam, Nigeria, China, Tanzania  * Srpska, China  * Yemen, Tanzania  * Peru | * Low to moderate  * Moderate  * Low  * Low to moderate  * Low to moderate  * Moderate  * Moderate |
| **Socio-demographic characteristics**  ***Parent education*** (conflicting findings):  * Higher educated parents less likely to give unprescribed antibiotics.  * Higher educated parents more likely to give unprescribed antibiotics.  ***Parent age*** (conflicting findings):  * Older parents/caregivers more likely to give unprescribed antibiotics.  * Younger parents more likely to give unprescribed antibiotics.  ***Child age*** *(conflicting findings):*  * Increasing age of child associated with unprescribed antibiotic use.  * Younger children more likely to be treated with antibiotics by the parent.  **Multiple household members:** | * [38]  * [32], [36]  * [41]  * [38]  * [51]  * [37], [48]  * [41], [51] | * Nigeria  * Vietnam  * China  * Nigeria  * China  * Bangladesh, Nigeria  * China | * Low  * Low  * Low  * Low  * Low  * Moderate  * Low |
| **Non-adherence to antibiotic treatment** | | | |
| **Themes and sub-themes** | **Studies** | **Location** | **Risk of bias** |
| **Parent-related determinants**  ***Attitudes and beliefs:***  * Child's symptoms improved  * Concern of potential harmful effects  * Lack of improvement in symptoms  ***Knowledge:***  * Low awareness of AMR and side effects  * Difficulties understanding the medicine regime.  ****Forgetting:*** | * [35], [41], [42], [51], [53], [54]  * [35], [54]  * [53]  * [34], [35], [40], [42]  * [20], [53]  * [20] | * Vietnam, China, Malawi, Bangladesh  * Vietnam, Bangladesh  * Malawi  * Vietnam, Yemen, China  * Australia, Malawi  * Australia | * Low  * Low  * Low  * Low to moderate  *Low  * Low |
| **External advice and influences**  **** Social and cultural norms:***  **** Palatability/child refusal:***  **** Lack of access to refrigeration to store antibiotic medicine:*** | * [53]  * [20]  * [20] | * Malawi  * Australia  * Australia | * Low  * Low  * Low |
| **Barriers to healthcare**  ***Greater distance to travel:*** | * [53] | * Malawi | * Low |
| **Requests for antibiotic prescriptions** | | | |
| **Themes and sub-themes** | **Studies** | **Location** | **Risk of bias** |
| **Parent-related determinants**  ***Attitudes and beliefs:***  *Attitudes it is appropriate to ask prescribers for antibiotics.  *Attitudes doctors should acquiesce to parental expectations for antibiotic prescriptions. | * [51]  * [47] | * China  * Peru | * Low  * Moderate |

**Note: †**Symptoms were associated with antibiotic use or parent expectations for antibiotics.
